# Prevalence of suicidal ideation among married and cohabiting women in Sri Lanka: An analysis of the Sri Lanka Women’s Well-being Survey 2019

**DOI:** 10.1371/journal.pone.0312753

**Published:** 2024-12-06

**Authors:** K. A. S. Thabrew, K. D. C. Ariyasena, S. A. H. M. Sandarapperuma, R. M. K. P. Weerasekara, M. T. S. Munasinghe, S. L. Ranamukhaarachchi, G. D. V. D. Wijayabandara

**Affiliations:** 1 Faculty of Science, Sri Lanka Technology Campus (PVT) Ltd, Padukka, Sri Lanka; 2 Centre for Big Data Research in Health, University of New South Wales, Sydney, Australia; 3 Department of Psychology, Sri Lanka Technology Campus (PVT) Ltd, Padukka, Sri Lanka; 4 Department of Statistics, Faculty of Science, University of Colombo, Colombo, Sri Lanka; 5 Faculty of Information Technology, University of Moratuwa, Moratuwa, Sri Lanka; 6 Faculty of Engineering and Technology, Sri Lanka Technology Campus (PVT) Ltd, Padukka, Sri Lanka; 7 The National Institute of Mental Health, Mulleriyawa New Town, Sri Lanka; University of Ghana College of Humanities, GHANA

## Abstract

This study utilized the 2019 Women’s Wellbeing Survey conducted by the Department of Census and Statistics (DCS) in Sri Lanka to investigate the factors influencing suicidal ideation among married/cohabiting women. The study sample consists of secondary data from 1462 females, who were 15 years or older and currently married or living with a male partner, extracted from WWS 2019. Binary logistic regression was employed to analyze the association between suicidal ideation in married/cohabiting women and various independent variables. Among the respondents, 13.2% of married/cohabiting women reported having suicidal thoughts. Several factors were found to increase the likelihood of such ideation: a partner’s extramarital affair, feelings of worthlessness and restlessness, partners consuming alcohol, experience of psychological violence and physical violence by the current partner, physical violence experienced since the age of 15, and childhood sexual violence. Conversely, lower odds of suicidal ideation were observed among women who married/cohabited between the ages of 20–29 compared to those in the 10–19 age category, and among unemployed women. This study emphasizes the urgency of addressing intimate partner violence, childhood sexual abuse, early marriages, and the mental well-being of vulnerable women, as these factors significantly impact their risk of life-threatening issues.

## Introduction

Suicide turns out to be a burning issue in most low–and middle–income countries. As per the statistics of the World Health Organization (WHO) in 2019, over 703,000 people died by suicide every year [1]. Suicide may occur during the first attempt itself, or there could be many suicidal attempts before a person departs from his life as a result.

Suicidal ideation, frequently referred to as suicidal thoughts or ideas, is a general term for a variety of thoughts, desires, and obsessions with ending one’s life [2]. Since there is no agreed-upon and uniform definition of suicidal ideation, researchers continue to face challenges in properly defining the idea. While some definitions of suicidal ideation include suicide planning considerations, others view planning as an entirely separate stage [2]. Therefore, according to some researchers, it is the self-reported contemplation of suicide-related conduct. Suicidal ideation is of two types namely, passive suicidal ideation and active suicidal ideation [3]. Passive suicidal ideation is defined as thoughts that are less overtly suicidal in nature, such as thoughts of wanting to die or not be awake. These ideas might also be perceived as ranging from passing notions that worthless of living to well-planned suicide plots to an intense, concern with self-destruction [4]. Active suicidal ideation refers to the condition where an individual experiences thoughts of dying by suicide or self-harm and has formulated a specific plan to execute it [4].

According to community surveys conducted by WHO in 21 countries, 12-month prevalence of suicidal ideation was approximately 2% and the lifetime prevalence was 9% [5, 6]. Research has also shown that suicidal attempts are significantly more common than suicide deaths. It has been reported that there are more than 30 suicide attempts for every suicide fatality in the United States each year [6]. As per the statistics from several research, the suicide mortality of males is higher than females; nevertheless, the lifetime prevalence of suicidal ideation is higher in females than in males [5, 7]. Additionally, there are two trends that constantly remain true as data analysis is broken down by gender. Nonfatal suicidal ideation by females is more common, but of males’ suicide rates are higher [7].

A previous study conducted in Sri Lanka found that the active suicidal ideation rate and passive suicidal ideation rate as 3.8% and 6.9%, respectively among females [8]. Authors of that study have observed the overall population, where 52% of the sample consisted of female participants and 68% of females in the total sample population were married/ever married. Another study conducted by Dutta *et al*. (2017) [9] in Sri Lanka, which looked into the genetic and other risk factors for suicidal ideation, showed that 21.8% of women were having suicidal ideation. Moreover, the pooled prevalence of overall suicidal ideation was 17% among women and girls in South Asia [10].

### Literature review

Many researchers have investigated suicidal ideation among general and specific populations in other countries. Kaplan *et al*. (1997) [11] conducted a study on the relationship between physical abuse during adolescence and suicide attempts. The results of that study demonstrated that abused adolescents were significantly more prone to risk factors related to adolescent suicide than those who have not been abused in their adolescence, family disintegration, depression, disruptive behavior disorders, and substance abuse and dependence. Moreover, it was found that women in Asia who were exposed to violence appear to have an increased risk of suicidal behavior and mental disorders [12–16]. In another study by Wikman *et al*. (2022) [17] a substantial proportion of women with confirmed premenstrual dysphoric disorder reported current suicidal ideation in the late luteal phase. Furthermore, Katzenmajer-Pump *et al*. (2022) [18] showed the importance of recognizing “worthlessness” for suicide prevention in adolescents with attention-deficit/hyperactivity disorder (ADHD). This study revealed that the symptoms of depression and anxiety mediate the relationship between ADHD and suicidal thoughts and planning. A study done in China examined the risk factors and clinical characteristics of suicidal ideation in Chinese patients with depression and the independent risk predictors included crying, helplessness, worthlessness, hopelessness, unusually restless, self-harm, mood-incongruent psychosis, feeling losing control of oneself, hypersomnia, sensory system complaints, derealization, guilt, suicidal attempts, male gender, the total course of depression [19]. Moreover, a study done in India found a significant positive association between the consumption of alcohol by husbands and the presence of depressive symptoms and suicidal thoughts in their wives [20]. Another study in India found that in contrast to married working women, single working women experience higher levels of suicidal thoughts. The same study indicated that, compared to working women between the ages of 46 and 55, women in the 35–43 age range reported more suicidal thoughts and the women employed in the private sector exhibited a higher rate of suicidal ideation compared to those in the public sector [21]. A study done by Kiliçarslan et al. (2024) determined several significant factors associated with the suicidal tendency of Turkish married women, women with a previous relationship, and women currently in a relationship, including age, educational level, health status, number of children, the sector in which the spouse or partner works, the drinking status of the spouse or partner, and household income level [22].

Several studies have been conducted on suicides and suicidal ideation among women in Sri Lanka where most existing literature has focused on the general population, youth, and/or pregnant women [8, 9, 23, 24]. In a study in 2006, sociological risk factors for youth suicidal behavior were found to include poor father-child interactions, low educational attainment, poor peer interaction, easy access to pesticides and medicinal drugs, a mother being away or abroad, breakups with romantic partners, and parents who exhibited alcoholism [23]. Genetic and other risk factors for suicidal ideation and the relationship with depression were examined in a study done in Sri Lanka [9] in which various factors were found to be associated with suicidal thoughts, such as being female, ending a marriage, low level of education, residing in an urban area, losing a parent at a young age, low standard of living, and experiencing stressful life events in the previous year, etc. The same study also found that suicidal ideation was closely related to depression, abnormal fatigue, and use of alcohol and tobacco. Furthermore, a significant impact of genetic and non-shared environmental factors on suicidal thoughts in both men and women was observed. For women, the genetic factor that contributes to suicidal ideation was mostly influenced by depression. However, in men, there was a distinct heritable component linked to suicidal thoughts that was not related to depression. Rajapakse *et al*. (2020) conducted a case-control analysis to investigate the connection between exposure to childhood adversity and hospital admissions for non-fatal self-poisoning in Sri Lanka. The results indicated that being physically or emotionally abused or neglected as a child, witnessing domestic violence, living with a family member who is mentally ill or suicidal, and experiencing parental death, separation, or divorce during childhood, etc. increased the chances of self-poisoning in adulthood [25]. Palfreyman (2021) studied the prevalence of common perinatal mental illnesses and suicidal tendencies among women residing in urban areas of Sri Lanka and the analysis revealed that the most significant factors associated with depression and suicidal ideation and/or behaviors during the current pregnancy were exposure to intimate partner violence and a history of suicidal ideation and/or behaviors [24]. Samaraweera *et al*. (2010) identified that the active suicidal ideators were high/frequent among physically ill women, and with higher levels of helplessness and hopelessness. Moreover, Bandara et al. (2022) found that women exposed to past-year domestic violence in Sri Lanka are 4.08 times more likely to self-harm than women not exposed to domestic violence [12]. In a study done by Bandara et al. (2024), suicide and intimate partner violence (IPV) were found to be correlated highlighting the importance of prioritizing trauma-informed approaches and addressing IPV in post conflict settings as suicide prevention initiatives in Sri Lanka [13]. Although these studies have explored factors associated with suicidal ideation in Sri Lanka, nearly all of them have utilized small sample sizes from specific locations or target groups. Furthermore, there have no studies examined the factors associated with suicidal ideation among married women or women cohabiting with a male partner in the Sri Lankan context.

This study aims to fill significant gaps in both local and global literature by examining, for the first time, the prevalence of suicidal ideation among married and cohabiting women in Sri Lanka, and identifying the factors associated with it. Utilizing a nationally representative large sample, this research ensures greater accuracy and provides novel insights into the subject. The originality of this study lies in its focus on a previously under-researched population, offering unique findings that can guide the development of targeted interventions. Furthermore, the results will be instrumental in identifying appropriate measures to reduce suicidal ideation in women vulnerable to sexual abuse, thereby offering valuable information to decision-makers, health authorities, and policymakers.

## Materials and methods

### Data source, sampling, and data collection

The data for this study were secondary data obtained on 26/10/2023 from the 2019 Women’s Well-being Survey (WWS) conducted in Sri Lanka by the Department of Census and Statistics (DCS). This was the first national-level survey conducted in Sri Lanka on violence against married and unmarried and cohabiting females [26]. The data were collected in this survey by using the Computer-Assisted Personal Interviewing Technique (CAPI) in the form of a questionnaire administered by female staff from the DCS. The WHO Multi-Country Study on Women’s Health and Life Experiences questionnaire version 12.04.01 (dated 10 July 2018) was used for this survey [26]. The survey used a multi-stage sampling approach, stratified by sector (urban, rural, and estate). The sampling frame used in the survey consisted of 65,000 census blocks, where 252 were selected as the primary sampling unit (PSU), and 10 housing units were selected from each selected PSU as the secondary sampling unit (SSU). The selection was done using a systematic random sampling method. Eligible women who were 15 years or older in each household were interviewed.

The study sample consists of secondary data from 1462 females, who were 15 years or older and currently married or living with a male partner, extracted from WWS 2019.

### Ethical approval

The survey was conducted by the DCS using the WHO’s ethical and safety recommendations for research on violence against women as a guide [26] and consent from each respondent was obtained before continuing. The detailed measures are given in the "Women’s Well-being Survey—2019 Final Report" published by the DCS Sri Lanka. Ethical approval is not required for this study as this is an analysis of secondary data where all data are anonymized by DCS to conceal the characteristics and the identity of the respondents. Therefore, the authors had no access to information that could identify individual participants during or after data collection.

### Variables

#### Dependent variable

During the survey, the respondents were asked the question "In your life, have you ever seriously thought about ending your life?" which is about suicidal ideation, and the expected answer was either "Yes" or "No." Hence, the variable "Ever had suicidal thoughts" was selected as the dependent variable, which is binary, with "Yes" coded as 1 and "No" coded as 0.

#### Independent variables

The study used 33 categorical independent variables, including socio-demographic variables such as the woman’s age, marital status, and highest education level, as well as variables indicating psychological distress symptoms such as depression, hopelessness, worthlessness, and experiences of physical, sexual, psychological violence, and economic violence by the women at different stages. Detailed descriptions of the independent variables are given in Table 1. The selection of independent variables was based on an analysis of relevant literature and the availability of those variables in the dataset considered.

**Table 1:** Details of independent variables.

Variable	Category	Scale of Measurement
Sector	Urban	Nominal
	Rural	
	Estate	
Marital Status	Married	Nominal
	Living Together	
Woman’s age (Years)	15 to 19	Ordinal
	20 to 29	
	30 to 39	
	40 to 49	
	50 to 59	
	60 or above	
Woman’s Highest Education Level	No Education	Ordinal
	Primary Education	
	Secondary Education	
	Higher Education	
Woman’s Working Status	Working	Nominal
	Not Working	
Main Source of Income	No Fixed Income	Nominal
	Money from Household Members Work	
	Support from Husband/ Partner	
	Support from Other Relatives	
	Pension	
	Social Services/ Welfare	
	Other Income Methods	
Can Count on Immediate Family Members when Needed	Yes	Nominal
	No	
Attitude on Overall Health	Excellent	Ordinal
	Good	
	Fair	
	Poor	
	Very Poor	
Presence of Any Health Problem	Yes	Nominal
	No	
Age of Woman at First Marriage or Union (Years)	10 to 19	Ordinal
	20 to 29	
	30 to 39	
	40 to 49	
Ever Given Birth	Yes	Nominal
	No	
Currently Living with Parents/ Relatives	Not Living with Husband’s or Own Parents	Nominal
	His Parents or Relatives	
	Her Parents or Relatives	
	With Both His and Her Parents or Relatives	
Current Husband’s/ Partner’s Highest Education Level	No Education	Ordinal
	Primary Education	
	Secondary Education	
	Higher Education	
Current Husband’s/ Partner’s Working Status	Working	Nominal
	Not Working	
Current Husband/ Partner Consumes Alcohol	Yes	Nominal
	No	
Current Husband/ Partner Consumes Drugs	Yes	Nominal
	No	
Current Husband/ Partner has an Affair with Another Woman	Yes	Nominal
	No	
	May Have	
How Often Quarrel with Current Husband/ Partner	Rarely	Ordinal
	Sometimes	
	Often	
	No	
Afraid of Current Husband/ Partner	Yes	Nominal
	No	
Faced Physical Violence by Current Husband/ Partner	Yes	Nominal
	No	
Faced Sexual Violence by Current Husband/ Partner	Yes	Nominal
	No	
Faced Psychological Violence by Current Husband/ Partner	Yes	Nominal
	No	
Faced Economical Violence by Current Husband/ Partner	Yes	Nominal
	No	
Faced Physical Violence from 15 years Old	Yes	Nominal
	No	
Faced Sexual Violence from 15 years Old	Yes	Nominal
	No	
Faced Physical Violence before 15 years Old	Yes	Nominal
	No	
Faced Sexual Violence before 15 years Old	Yes	Nominal
	No	
Feeling Depressed	None of the time	Ordinal
	A little of the time	
	Often	
Feeling Hopeless	None of the time	Ordinal
	A little of the time	
	Often	
Feeling Worthless	None of the time	Ordinal
	A little of the time	
	Often	
Feeling Nervous	None of the time	Ordinal
	A little of the time	
	Often	
Feeling Restless	None of the time	Ordinal
	A little of the time	
	Often	
Feeling That Everything was an Effort	None of the time	Ordinal
	A little of the time	
	Often	

### Statistical analysis

A bivariate analysis was performed to investigate the association between each independent variable and the dependent variable using the chi-square test of association. Variables that were significant at 5% in the chi-square test of association were selected to fit a binary logistic regression model with backward elimination to identify the effect of the association between the dependent variable and the selected independent variables, as well as the most contributing risk factors of suicidal ideation. The odds ratios were then interpreted for the significant variables. The performance of the final model was checked using a receiver operating characteristic (ROC) curve and the area under the curve (AUC). Finally, the model assumptions were tested. Data analysis was performed using Statistical Analysis System (SAS) On Demand for Academics. Moreover, p < 0.05 was considered the acceptable statistical level of significance, and 95% confidence intervals (CI) were used for the odds ratio.

## Results

### Baseline characteristics

The baseline characteristics of the study sample are shown in Table 2. Of the 1,462 study participants, 13.2% (193) of the married or cohabiting women reported having suicidal ideation. Although a predominant number of women lived in rural areas (79.69%) at the time of the survey, the highest prevalence of suicidal ideation was found in the estate sector (18.75%). By the time of the survey, 99.59% of the women in the sample were married, and the highest prevalence of suicidal ideation was observed among women who lived together with a partner (83.33%). Additionally, 25.1% of the study sample consisted of women aged between 40 and 49 years, and 79.48% of women had up to secondary education. Women aged between 40–49 years and women with no education reported the highest prevalence of suicidal ideation (17.71% and 27.27%, respectively). While 67.03% of women were not working at the time of the survey, 70.31% of women had their household income coming from household members’ work. Moreover, the highest prevalence of suicidal ideation was observed among working women (18.05%) and women with no fixed income (23.08%). Of the sample, 21.47%, 21.00%, 22.09%, 15.19%, 19.84%, and 27.57% of women reported feeling nervous, restless, depressed, worthless, hopeless, and that everything was an effort, often. The prevalence of suicidal ideation was also highest among those who often felt likewise (26.75%, 32.57%, 31.89%, 33.33%, 32.07%, and 26.55%, respectively). Moreover, 26.2%, 17.85%, 6.02%, and 15.05% of respondents reported that they had faced psychological, physical, sexual, and economical violence from their current husband or partner, respectively. The highest prevalence of suicidal ideation was reported among women who had faced psychological, physical, sexual, and economical violence from their current husband or partner (29.77%, 39.85%, 47.73%, and 25.91%, respectively). Furthermore, 7.66% and 25.44% of women experienced physical violence since they were 15 years old and before 15 years old, respectively. The prevalence of suicidal ideation was highest among those who had encountered physical violence (33.04% and 16.67%, respectively). Additionally, 3.76% and 1.71% of women experienced sexual violence since 15 years old and before 15 years old, respectively, and they reported the highest prevalence of suicidal ideation (23.64% and 48.00%, respectively). Of the respondents, 82.63% reported that they could count on the members of their birth family for support, and the highest prevalence of suicidal ideation was observed among women who reported otherwise (18.90%). Additionally, more than half of the women in the study sample had their first marriage or union with a man during 20 to 29 years of age (63.47%). The prevalence of suicidal ideation was highest among women who had their first marriage or union with a man during 10 to 19 years of age (20.63%). Of the sample, 72.09% were found independent and not living with their parents, in-laws, or relatives. Those who lived with their parents, in-laws, or relatives reported the highest prevalence of suicidal ideation (37.50%). Moreover, 64.57% of women reported health problems such as weak vision, hearing, mobility, memory, communication, etc., while the rest reported no health issues. The prevalence of suicidal ideation was highest among those who reported having health problems (14.94%). Additionally, 92.89% of women had given birth to a child, and the prevalence of suicidal ideation was highest among them (13.62%). Of the sample, 77.43% and 87.55% of women had husbands/partners with secondary education and employment, respectively. Women living with husbands/partners with no education and those who were with working husbands/partners reported the highest prevalence of suicidal ideation (21.21% and 13.28% respectively). It was also mentioned that 56.57% and 2.8% of their husband/ partner consumed alcohol and drugs, respectively, and the prevalence of suicidal ideation was also high among them (17.41% and 36.59%, respectively). Around 4% of women confirmed that their current husband/ partner had an affair with another woman, and suicidal ideation prevalence was also high among them (52.73%). Moreover, 3.9% reported often quarreling with their current husband or partner, and they reported the highest prevalence of suicidal ideation as well (50.88%).

**Table 2:** Bivariate analysis of dependent and independent variables.

Variable	Category	Overall (n = 1462)	Suicidal Ideation		P-Value
			No (n = 1269)	Yes (n = 193)	
		N(%)	N(%)	N(%)	
Sector	Urban	233 (15.94)	209 (89.70)	24 (10.30)	0.1731
	Rural	1,165 (79.69)	1,008 (86.52)	157 (13.48)	
	Estate	64 (04.38)	52 (81.25)	12 (18.75)	
Marital Status	Married	1,456 (99.59)	1,268 (87.09)	188 (12.91)	<0.0001***
	Living Together	6 (00.41)	1 (16.67)	5 (83.33)	
Woman’s age (Years)	15 to 19	3 (00.21)	3 (100.00)	0 (00.00)	0.0266*
	20 to 29	143 (09.78)	133 (93.01)	10 (06.99)	
	30 to 39	361 (24.69)	313 (86.70)	48 (13.30)	
	40 to 49	367 (25.10)	302 (82.29)	65 (17.71)	
	50 to 59	313 (21.41)	275 (87.86)	38 (12.14)	
	60 or above	275 (18.81)	243 (88.36)	32 (11.64)	
Woman’s Highest Education Level	No Education	55 (03.76)	40 (72.73)	15 (27.27)	0.0008***
	Primary Education	175 (11.97)	144 (82.29)	31 (17.71)	
	Secondary Education	1,162 (79.48)	1,019 (87.69)	143 (12.31)	
	Higher Education	70 (04.79)	66 (94.29)	4 (05.71)	
Woman’s Working Status	Working	482 (32.97)	395 (81.95)	87 (18.05)	0.0001***
	Not Working	980 (67.03)	874 (89.18)	106 (10.82)	
Main Source of Income	No Fixed Income	52 (03.56)	40 (76.92)	12 (23.08)	0.2386
	Money from Household Members’ Work	1,028 (70.31)	890 (86.58)	138 (13.42)	
	Support from Husband/ Partner	306 (20.93)	272 (88.89)	34 (11.11)	
	Support from Other Relatives	17 (01.16)	14 (82.35)	3 (17.65)	
	Pension	46 (03.15)	42 (91.30)	4 (08.70)	
	Social Services/ Welfare	4 (00.27)	4 (100.00)	0 (00.00)	
	Other Income Methods	9 (00.62)	7 (77.78)	2 (22.22)	
Can Count on Immediate Family Members when Needed	Yes	1,208 (82.63)	1,063 (88.00)	145 (12.00)	0.0032**
	No	254 (17.37)	206 (81.10)	48 (18.90)	
Attitude on Overall Health	Excellent	369 (25.24)	334 (90.51)	35 (09.49)	0.0046**
	Good	552 (37.76)	488 (88.41)	64 (11.59)	
	Fair	390 (26.68)	325 (83.33)	65 (16.67)	
	Poor	136 (09.30)	109 (80.15)	27 (19.85)	
	Very Poor	15 (01.03)	13 (86.67)	2 (13.33)	
Presence of Any Health Problem	No	518 (35.43)	466 (89.96)	52 (10.04)	0.0081**
	Yes	944 (64.57)	803 (85.06)	141 (14.94)	
Age of Woman at First Marriage or Union (Years)	10 to 19	383 (26.20)	304 (79.37)	79 (20.63)	<0.0001***
	20 to 29	928 (63.47)	830 (89.44)	98 (10.56)	
	30 to 39	142 (09.71)	126 (88.73)	16 (11.27)	
	40 to 49	9 (00.62)	9 (100.00)	0 (00.00)	
Ever Given Birth	No	104 (07.11)	96 (92.31)	8 (07.69)	0.0851
	Yes	1,358 (92.89)	1,173 (86.38)	185 (13.62)	
Currently Living with Parents/ Relatives	Not Living with Husband’s or Own Parents	1,054 (72.09)	909 (86.24)	145 (13.76)	0.0545
	His Parents or Relatives	263 (17.99)	229 (87.07)	34 (12.93)	
	Her Parents or Relatives	137 (09.37)	126 (91.97)	11 (08.03)	
	With Both His and Her Parents or Relatives	8 (00.55)	5 (62.50)	3 (37.50)	
Current Husband’s/ Partner’s Highest Education Level	No Education	33 (02.26)	26 (78.79)	7 (21.21)	0.0390*
	Primary Education	228 (15.60)	197 (86.40)	31 (13.60)	
	Secondary Education	1,132 (77.43)	979 (86.48)	153 (13.52)	
	Higher Education	69 (04.72)	67 (97.10)	2 (02.90)	
Current Husband’s/ Partner’s Working Status	Working	1,280 (87.55)	1,110 (86.72)	170 (13.28)	0.8102
	Not Working	182 (12.45)	159 (87.36)	23 (12.64)	
Current Husband/ Partner Consumes Alcohol	Yes	827 (56.57)	683 (82.59)	144 (17.41)	<0.0001***
	No	635 (43.43)	586 (92.28)	49 (07.72)	
Current Husband/ Partner Consumes Drugs	Yes	41 (02.80)	26 (63.41)	15 (36.59)	<0.0001***
	No	1,421 (97.20)	1,243 (87.47)	178 (12.53)	
Current Husband/ Partner has an Affair with Another Woman	Yes	55 (03.76)	26 (47.27)	29 (52.73)	<0.0001***
	No	1,397 (95.55)	1,236 (88.48)	161 (11.52)	
	May Have	10 (00.68)	7 (70.00)	3 (30.00)	
How Often Quarrel with Current Husband/ Partner	Rarely	577 (39.47)	505 (87.52)	72 (12.48)	<0.0001***
	Sometimes	231 (15.80)	183 (79.22)	48 (20.78)	
	Often	57 (03.90)	28 (49.12)	29 (50.88)	
	No	597 (40.83)	553 (92.63)	44 (07.37)	
Afraid of Current Husband/ Partner	Yes	1,142 (78.11)	1,031 (90.28)	111 (09.72)	<0.0001***
	No	320 (21.89)	238 (74.38)	82 (25.63)	
Faced Physical Violence by Current Husband/ Partner	Yes	261 (17.85)	157 (60.15)	104 (39.85)	<0.0001***
	No	1,201 (82.15)	1,112 (92.59)	89 (07.41)	
Faced Sexual Violence by Current Husband/ Partner	Yes	88 (06.02)	46 (52.27)	42 (47.73)	<0.0001***
	No	1,374 (93.98)	1,223 (89.01)	151 (10.99)	
Faced Psychological Violence by Current Husband/ Partner	Yes	383 (26.20)	269 (70.23)	114 (29.77)	<0.0001***
	No	1,079 (73.80)	1,000 (92.68)	79 (07.32)	
Faced Economical Violence by Current Husband/ Partner	Yes	220 (15.05)	163 (74.09)	57 (25.91)	<0.0001***
	No	1,242 (84.95)	1,106 (89.05)	136 (10.95)	
Faced Physical Violence from 15 years Old	Yes	112 (07.66)	75 (66.96)	37 (33.04)	<0.0001***
	No	1,350 (92.34)	1,194 (88.44)	156 (11.56)	
Faced Sexual Violence from 15 years Old	Yes	55 (03.76)	42 (76.36)	13 (23.64)	0.0198*
	No	1,407 (96.24)	1,227 (87.21)	180 (12.79)	
Faced Physical Violence before 15 years Old	Yes	372 (25.44)	310 (83.33)	62 (16.67)	0.0222*
	No	1,090 (74.56)	959 (87.98)	131 (12.02)	
Faced Sexual Violence before 15 years Old	Yes	25 (01.71)	13 (52.00)	12 (48.00)	<0.0001***
	No	1,437 (98.29)	1,256 (87.40)	181 (12.60)	
Feeling Depressed	None of the time	962 (65.80)	892 (92.72)	70 (07.28)	<0.0001***
	A little of the time	177 (12.11)	157 (88.70)	20 (11.30)	
	Often	323 (22.09)	220 (68.11)	103 (31.89)	
Feeling Hopeless	None of the time	1,031 (70.52)	953 (92.43)	78 (07.57)	<0.0001***
	A little of the time	141 (09.64)	119 (84.40)	22 (15.60)	
	Often	290 (19.84)	197 (67.93)	93 (32.07)	
Feeling Worthless	None of the time	1,099 (75.17)	1,012 (92.08)	87 (07.92)	<0.0001***
	A little of the time	141 (09.64)	109 (77.30)	32 (22.70)	
	Often	222 (15.19)	148 (66.67)	74 (33.33)	
Feeling Nervous	None of the time	964 (65.94)	885 (91.80)	79 (08.20)	<0.0001***
	A little of the time	184 (12.59)	154 (83.70)	30 (16.30)	
	Often	314 (21.47)	230 (73.25)	84 (26.75)	
Feeling Restless	None of the time	979 (66.96)	909 (92.85)	70 (07.15)	<0.0001***
	A little of the time	176 (12.04)	153 (86.93)	23 (13.07)	
	Often	307 (21.00)	207 (67.43)	100 (32.57)	
Feeling That Everything was and Effort	None of the time	892 (61.01)	830 (93.05)	62 (06.95)	<0.0001***
	A little of the time	167 (11.42)	143 (85.63)	24 (14.37)	
	Often	403 (27.57)	296 (73.45)	107 (26.55)	

Level of significance:

* p < 0.05,

** p < 0.01,

*** p < 0.001

### Effect of associated factors on suicidal ideation

The adjusted odds ratios (AOR) were calculated to determine the strength of the association between suicidal ideation among married/ cohabiting women and the independent variables that remained in the final model after adjusting for all variables in the final model (Table 3). It was found that a woman’s working status had a significant association, with unemployed women having 50% reduced odds of suicidal ideation (AOR: 0.504; 95% CI: 0.347–0.732] compared to working women. As expected, the odds of suicidal ideation were almost three times higher for women who reported that their current husband or partner had an affair with another woman (AOR: 2.909; 95% CI: 1.456–5.812) compared to women with partners who did not report infidelity. In addition, the odds of suicidal ideation were three and two times higher for women who reported feeling restless (AOR: 3.107; 95% CI: 1.936–4.986) and worthless (AOR: 2.44; 95% CI: 1.484–4.014), respectively, often when compared to women who reported not having such feelings at any time. The odds of suicidal ideation were almost 60% higher among women whose husbands or partners consumed alcohol (AOR: 1.595; 95% CI: 1.073–2.371) compared to women who had a teetotaler. A significant association was observed between psychological and physical violence by the current husband or partner and suicidal ideation, where the odds of suicidal ideation were two and four times higher among women who faced psychological violence (AOR: 2.090; 95% CI: 1.405–3.109) and physical violence (AOR: 3.794; 95% CI: 2.501–5.754), respectively, from the current husband or partner. Moreover, the odds of suicidal ideation were 72% higher among women who encountered physical violence from age 15 until the study (AOR: 1.722; 95% CI: 1.001–2.964) compared to women with no such encounters. A highly significant association between suicidal ideation and childhood sexual violence was observed. (AOR: 4.721; 95% CI: 1.851–12.046). Odds of having suicidal ideation of women who got married/ started living together with a man at an age between 20–29 years were less when compared with 10–19 age category (AOR of 0.563; 95% CI: 0.381, 0.832) and this shows the protective effect of marrying at a later age compared to younger age.

**Table 3:** Odds ratio estimates.

Effect	Point Estimate	95% CI ^a^	P—value
Working Status: Not Working	0.504	0.347–0.732	0.0003***
ref (Working)			
Feeling Restless: A little of the time	1.240	0.671–2.293	0.4922
Feeling Restless: Often	3.107	1.936–4.986	<0.0001***
(ref: None of the time)			
Feeling Worthless: A little of the time	2.361	1.323–4.215	0.0037**
Feeling Worthless: Often	2.440	1.484–4.014	0.0004***
(ref: None of the time)			
Age of Woman at First Marriage or Union (Years): 20 to 29	0.563	0.381–0.832	0.0039**
Age of Woman at First Marriage or Union (Years): 30 to 39	0.723	0.371–1.411	0.3422
Age of Woman at First Marriage or Union (Years): 40 to 49	<0.001	<0.001–>999.999	0.9859
(ref: 10 to 20)			
Current Husband/ Partner Consumes Alcohol: Yes	1.595	1.073–2.371	0.0210*
(ref: No)			
Current Husband/ Partner has an Affair with Another Woman: Yes	2.909	1.456–5.812	0.0025**
Current Husband/ Partner has an Affair with Another Woman: May Have	0.570	0.108–3.011	0.5079
(ref: No)			
Faced Psychological Violence by Current Husband/ Partner: Yes	2.090	1.405–3.109	0.0003***
(ref: No)			
Faced Physical Violence by Current Husband/ Partner: Yes	3.794	2.501–5.754	<0.0001***
(ref: No)			
Faced Physical Violence from 15 years Old: Yes	1.722	1.001–2.964	0.0497*
(ref: No)			
Faced Sexual Violence before 15 years Old: Yes	4.721	1.851–12.046	0.0012**
(ref: No)			

^a^ 95% CI: 95% Confidence Interval

Level of significance:

* p < 0.05,

** p < 0.01,

*** p < 0.001

**Fig 1:**
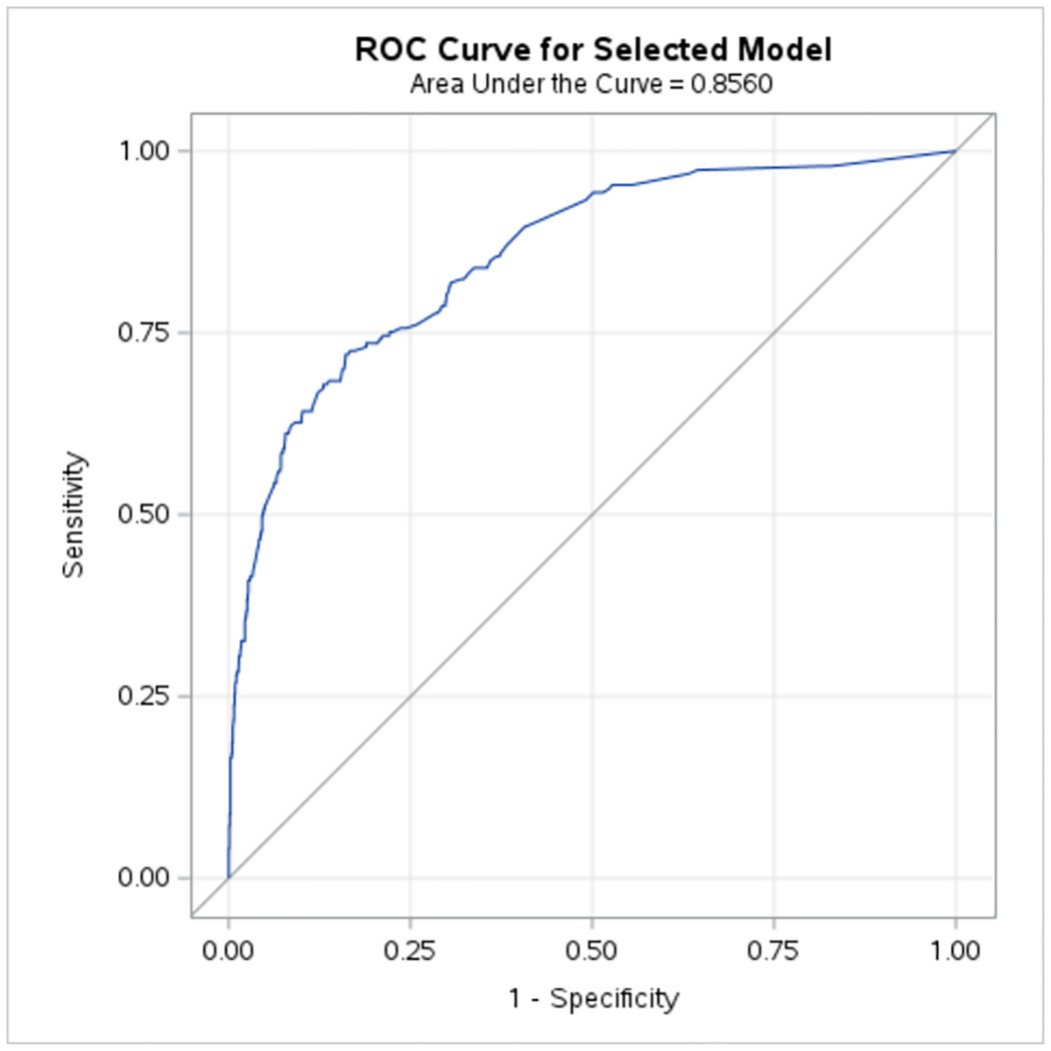
Receiver operating characteristic (ROC) curve for logistic model.

Fig 1 demonstrates the performance of the fitted logistic regression model for predicting suicidal ideation among Sri Lankan women who were married or living with a man. The AUC of the logistic regression model was 0.8560 (95% CI: 0.8265, 0.8855), which indicated a very good predictive ability.

### Model assumptions

The assumption of no multicollinearity among the independent variables was tested using the variance inflation factor (VIF). The results showed a mean VIF value of 1.5, indicating the absence of high multicollinearity among the independent variables. The assumption of independence of errors was tested for overdispersion by calculating the ratio of deviance and Pearson statistics values to the degrees of freedom of the final model. The ratio of deviance (0.59) and the ratio of Pearson statistic (1.00), both being less than 1, indicated no overdispersion. The linearity assumption in logistic regression implies that each continuous predictor is linearly related to the logit of the outcome [27]. Since all the variables are categorical, this assumption did not need to be tested. Finally, the presence of outliers was checked by examining the standardized residuals between the predicted and observed outcomes. Residuals greater than 3 or less than -3 are considered potential outliers [28]. In this study, out of 1462 cases, 43 outliers were identified.

## Discussion

The present study examined the prevalence and associated factors of suicidal ideation among married/cohabiting women in Sri Lanka using the 2019 WWS conducted in Sri Lanka. The study included 1,462 married/cohabiting women. Results showed that overall, 13 out of 100 married or cohabiting women in Sri Lanka reported having suicidal ideation.

Although many previous local and global studies [23, 29–32] have shown that suicidal ideation is associated with unemployment, the results of this study revealed that unemployed women have 50% reduced odds of suicidal ideation compared to working women. The reasons for this difference may be attributed to the sample’s characteristics and thus requiring further analysis. However, research done in India has shown the same results indicating that employed women who are unmarried are more prone to higher levels of suicidal thoughts [21].

Suicidal ideation was observed to be high among women who reported that their intimate partner has extramarital affairs or a relationship with another woman while being with the study participant. The person who has been harmed may experience various emotions such as anger, helplessness, abandonment, and feeling like a victim, which can be intense and unpredictable. These feelings can leave them vulnerable and incapable of managing their own well-being after discovering the own partner has been unfaithful. This could potentially lead to thoughts of self-harm or suicide [33]. Many global studies have revealed similar results, showing that infidelity by an intimate partner is associated with suicidal thoughts [22, 33–39].

Restlessness has been found to be an associated factor with suicidal ideation in many previously conducted studies [19, 22, 40, 41]. The results of the current study also yielded similar results. While it is normal to experience restlessness at times, frequent episodes of restlessness could indicate the presence of psychiatric conditions such as depression, bipolar disorder, ADHD, and dementia [42], and which are associated with suicidal thoughts [43].

The results of the present study also revealed that the feeling of worthlessness often by women is associated with a higher likelihood of suicidal ideation. These findings are supported by studies from India, UK, Japan, Sweden, and China [17, 19, 44–46]. Furthermore, studies have shown that the feeling of worthlessness was a primary contributor to suicidal ideation [18].

A current study has shown that the odds of suicidal ideation were low among women who got married or started cohabiting at an age between 20–29 years as opposed to 10–19 years age category. 10–19 years is typically considered underage for marriage in many countries in the world [47]. Furthermore, underage marriage and child marriage or union with a man at a very young age were found to be often associated with suicidal ideation in many of the global studies [39, 48–52]. These studies have shown that suicidal ideation among women who got married or started living together with a man in their childhood or adolescence, is fueled by the negative marital circumstances, lack of health, lack of care, unwanted pregnancies and overburden with domestic work which stimulate to depressive symptoms and many mental disorders. Newly married adolescent women in South Asia (including Sri Lanka) often leave their birth relatives and are required to move into the household of their spouse, frequently with their in-laws. There is previous evidence in Sri Lanka which shows that women who live with their in-laws are more likely to experience domestic violence, by any household member [14]. It is possible that the significant change in lifestyle circumstances, social connections, and new expectations placed on adolescent woman place new challenges and stress on the young woman, increasing likelihood for suicidal ideation.

Previously studies conducted locally and globally have revealed that an intimate partner’s alcohol consumption is associated with suicidal ideation among women [20, 22, 24, 53–55]. The findings of this study are also in agreement with the aforementioned finding and showed that the likelihood of suicidal ideation among married/ cohabiting women increases with the intimate partner being alcoholic. Wives of alcoholic men may exhibit more symptoms of depression [56], experience intense feelings of unhappiness, frequent suicidal ideations [20] compared to those of non- alcoholic men.

The current study found that the odds of suicidal ideation were two and four times higher among women who reported facing psychological and physical violence, respectively, from their husbands or partners. The results agree with previous studies conducted in Sri Lanka [24, 57]. Moreover, studies conducted in four South Asian countries and China have also found a higher likelihood of suicidal ideation among women who have encountered psychological and physical violence by their intimate partner [58–63]. Research conducted in Afghanistan and India similarly showed significant links between lifetime exposure to domestic violence and self-harm [61, 64]. A significant number of women who has experienced violence and attempted suicide has been diagnosed with mental health conditions such as depression, anxiety, and post-traumatic stress disorder [65].

The present study also found that women who experienced physical violence from the age of 15 onward have a 72% higher likelihood of suicidal ideation than women who did not have such encounters. This finding is supported by a study by Kaplan et al [11] who investigated the impact of adolescent physical abuse on suicidal attempts. However, no study has examined the association between physical violence from age 15 and suicidal ideation among women in Sri Lanka, highlighting the need for further research in this area. Hence further research is needed on this aspect.

Previous studies have revealed, in Sri Lanka, that adverse childhood experiences, including sexual abuse, were associated with suicidal ideation and attempts among young and adult women [25, 66]. The results of studies conducted in Bangladesh, the UK, and the USA are also in agreement with the findings of the present study [60, 67–69]. It is suggested that childhood sexual abuse may cause decreased self-esteem, feelings of guilt and self-blame, a sense of powerlessness, and difficulties with relationships, all of which can contribute to a higher likelihood of developing self-destructive behaviors in women [70]. According to the results of this study, the likelihood of suicidal ideation was higher among women who experienced sexual violence in childhood compared to those who did not.

The factors that were considered significant in the reviewed literature [8, 9, 60], such as education level, household income source, sector, feelings of depression, marital status, helplessness, hopelessness, and presence of health problems did not show statistical evidence of an association in the current study based on the available data. Although the statistical model did not show statistical evidence of an association, a relatively high prevalence of suicidal ideation was found among women with no education and women whose partner had no education and women with no fixed income. These show a sign of a socioeconomic gradient, whereby lower educational attainment and no fixed income were associated with a higher prevalence of suicidal ideation and this prevalence decreased with higher education. Women who had no education, and women married or partnered with someone with no education had a higher prevalence of suicidal ideation. The prevalence of suicidal ideation decreased with higher educational attainment. This suggests that higher education of men and women has a protective effect on suicidal ideation. Moreover, higher prevalence of suicidal ideation was present among women from the estate sector, and among women experiencing physical and psychological health problems.

### Limitations of the study

This study is a cross-sectional study as it only used data from the 2019 WWS Sri Lanka. As a result, causal inference between the predictor variables and suicidal ideation cannot be considered. The study utilized variables that were significant and considered in previous literature that were also available in the study dataset. However, other factors that may be associated with suicidal ideation and that were not included in the dataset were not considered. Another limitation is that the observations in the dataset were limited, but there were many explanatory variables, which could lead to over-fitting of the models and incorrect predictions. Furthermore, the information provided in the study was based on self-reported data, which carries the risk of being unreliable or deceptive, and there is also a possibility of recall bias being present.

### Implications for practice and/or policy

The findings of the current study found a strong link between depression symptoms such as worthlessness, restlessness and suicidal ideation in married or cohabiting women in Sri Lanka. Successful psychological, pharmacological, and/or social intervention can lead to a decrease in suicidal thoughts. Unfortunately, many women in Sri Lanka are hesitant to seek mental health services due to their stigma and discrimination, which can exacerbate their mental health problems and increase the risk of suicide.

It is crucial to encourage women to seek help when necessary and address the stigma associated with mental health services in society. In addition, authorities should implement measures to decrease intimate partner violence and, physical and sexual abuse during childhood and adolescence, educate parents, teachers, and school children about various types of abuse and the process of taking appropriate legal actions to prevent recurrence. The authorities should also provide support to victims to recover from the psychological and physical trauma caused by such abuse; hence, many stakeholders need to be involved in managing such complicated cases. It is crucial to promote relationship counseling for couples as a mean to enhance the bond between women and their intimate partners.

## Conclusions

The current study investigated the prevalence of suicidal ideation in married and cohabiting women in Sri Lanka and the factors that may contribute to it. The results revealed that employed women, those experiencing restlessness and worthlessness, women with alcoholic or thought to be unfaithful partners, those who have faced physical or sexual violence from their intimate partner, or those who experienced childhood sexual abuse were more likely to experience suicidal ideation. These findings could help authorities and society at large to develop strategies to reduce intimate partner violence, childhood sexual abuse, prevent underage marriages and cohabitation, improve mental health, and promote positive relationships between women and their intimate partners.

## Abbreviations

ADHD: Attention-Deficit/Hyperactivity Disorder
AUC: Area Under the Curve
CAPI: Computer-Assisted Personal Interviewing Technique
CI: Confidence Intervals
DCS: Department of Census and Statistics
PSU: Primary Sampling Unit
ROC: Receiver Operating Characteristic
SAS: Statistical Analysis System
SSU: Secondary Sampling Unit
VIF: Variance Inflation Factor
WHO: World Health Organization
WWS: Women’s Well-being Survey
